# Correlation of pelvic incidence with radiographical parameters for acetabular retroversion: a retrospective radiological study

**DOI:** 10.1186/s12880-015-0080-1

**Published:** 2015-09-29

**Authors:** Simon Tiziani, Lucienne Gautier, Jan Farei-Campagna, Georg Osterhoff, Thorsten Jentzsch, Thi Dan Linh Nguyen-Kim, Clément ML Werner

**Affiliations:** Department of Surgery, Division of Trauma Surgery, University Hospital Zurich, Raemistrasse 100, 8091 Zurich, Switzerland; Institute of Diagnostic and Interventional Radiology, University Hospital Zurich, Raemistrasse 100, 8091 Zurich, Switzerland

**Keywords:** Pelvic incidence, Acetabular retroversion, Posterior wall sign, Cross-over sign, Prominence of the ischial spine sign, COS, PRISS, PWS

## Abstract

**Background:**

Pelvic incidence (PI) has been linked to several degenerative processes within the spinopelvic system. Acetabular retroversion is a recognised risk factor for osteoarthritis of the hip. We therefore hypothesised that these two factors might be part of a specific anatomical variant associated with degenerative changes. This study was performed to clarify this issue.

**Methods:**

The pelvic incidence was measured on 589 computertomographical data sets acquired between 2008 and 2010. For 220 patients a 2D rendering in an antero-posterior view of the CT data set was performed to evaluate the parameters of acetabular retroversion. Those included the prominence of the ischial spine sign (PRISS), the cross-over sign (COS) and the posterior wall sign (PWS). Between 477 and 478 hips were evaluated depending on the parameter of retroversion.

**Results:**

The mean pelvic incidence was significantly lower in hips positive for the PRISS and the PWS. However, there were no significant differences between hips positive or negative for the COS.

**Discussion:**

As hypothesised, the lower PI values in PWS and PRISS positive hips suggest a link between PI and retroversion of the acetabulum. Whether this is of any clinical relevance remains, however, unknown.

**Conclusion:**

Acetabular retroversion is linked to PI. In hips where the prominence of the ischial spine sign and/or the posterior wall sign was present, the mean pelvic incidence value was lower.

## Background

The acetabulum in a human pelvis is anteverted. In a normal asymptomatic population the mean anteversion is around twenty degrees [1]. Retroverted acetabula have been associated with dysplasia of the hip and are considered a risk factor for femoral hip pain, impingement and osteoarthritis [2–7]. Furthermore, the degree of anteversion or retroversion is believed to contribute to the pattern in acetabular fractures and occurrence in stress fractures of the femoral head [8, 9]. There are several radiographical markers to identify and quantify acetabular retroversion in a.p. radiographs. These include the cross-over sign (COS), the posterior wall sign (PWS) and the prominence of the ischial spine sign (PRISS). The coexistence of the COS with the PRISS and/or the PWS is usually associated with increasing severity of the acetabular retroversion [10–12].

During the last ten years, pelvic incidence (PI) has been established as a parameter for pelvic orientation. Legaye et al. first described PI as a parameter for pelvic configuration independent from pelvic movement [13]. PI is the sum of the sacral slope and the pelvic tilt. Multiple studies analysed PI in normal healthy populations and compared them to patients with specific disorders. It was found that PI is a hugely variable parameter in normal healthy adults, with a mean value of around fifty degrees and increasing with advancing age [14–17]. There have been several studies linking PI to spinopelvic disorders such as spondylolisthesis and osteoarthritis of the hip (HAO). An increase in PI was seen in a group of patients with HOA compared to patients with lower back pain [18–20]. In addition, a strong association between PI and spinal parameters was discovered [21]. PI affects spinal orientation and curvature and correlates directly with lumbar lordosis (LL) [17]. An increase in PI is often accompanied by an increase in LL. It is believed that with increasing PI and an associated development of a more pronounced LL, an individual has a wider range of motion regarding pelvic flexion around the bicoxofemoral axis, compared to subjects with low PI and little LL. Thus, our hypothesis was that those subjects with lower PI have limited possibilities to compensate stresses within the spinopelvic system, resulting in pathological loading and consequently acetabular retroversion. A correlation between a radiological marker and acetabular retroversion that is recognised to be a risk factor for osteoarthritis would allow early detection of patients at risk for osteoarthritis of the hip due to a lack of compensatory capabilities at the spinopelvic transition. The hypothesis was that PI correlates with acetabular retroversion in the way that, with decreasing PI, the probability for acetabular retroversion increases as well. The aim of this study was to clarify this issue.

## Methods

Between 2008 and 2010, 589 patients (191f/ 398 m, aged 14–94) underwent a computer tomography (CT) of the abdomen (ranging from the bottom of the lung, including the diaphragm, down to the trochanter minor) during their hospitalization, where the pelvic incidence could be measured. As there was often no conventional anterio-posterior radiograph taken because a CT was found to be sufficient, it was necessary to process a 2D anterio-posterior view (a.p. view) from the acquired CT data to evaluate the parameters of acetabular retroversion described for anterio-posterior radiographs using dedicated a software, VOXAR™ (TMVS Europe, Edinburgh, UK). All reconstructions were done in accordance to the criteria set forth by Siebenrock et al. [22]. 220 CT data sets were converted to anterio-posterior views of the pelvis using this method. Exclusion criteria included osseous lesions, osteosynthesis, or prosthesis of the hip.

### Pelvic incidence (Fig. 1)

**Fig. 1:**
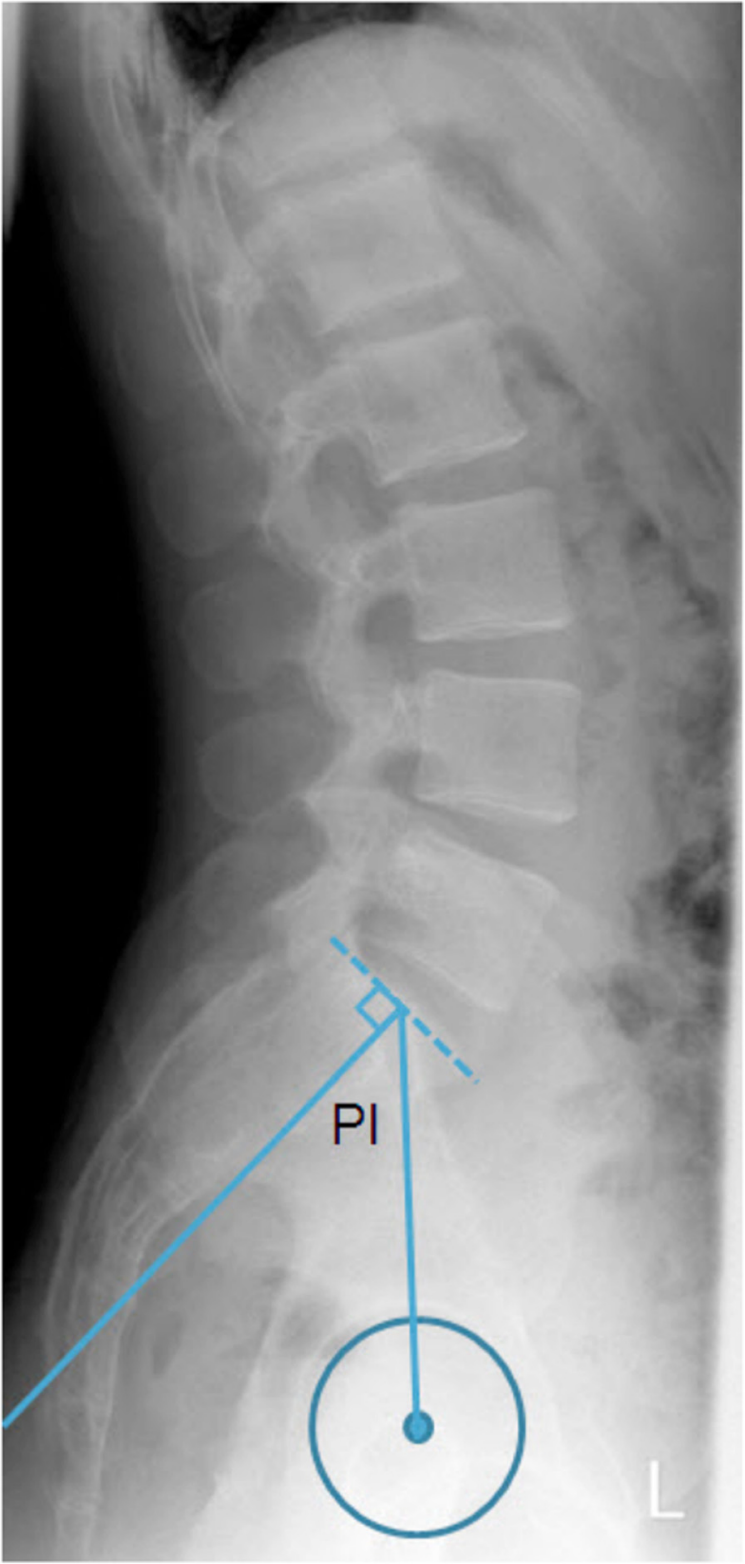
Lateral view of the lower spine and pelvis showing the pelvic incidence angle

The PI is the sum of the pelvic tilt and the sacral slope. It is measured by finding the mid-point between the femoral heads, followed by measuring the angle between the line from this midpoint to the middle of the upper edge of S1 in the sagittal view, and a line perpendicular to the upper edge of S1 [21]. In all patients, the PI was measured using CT scans of the abdomen. For this reason PI had to be calculated as the mean of the angles resulting from connecting the middle of the upper edge of S1 and the centres of the femoral heads on both sides rather then the mid-point of their joining line.

### Cross-over sign (Fig. 2b)

**Fig. 2:**
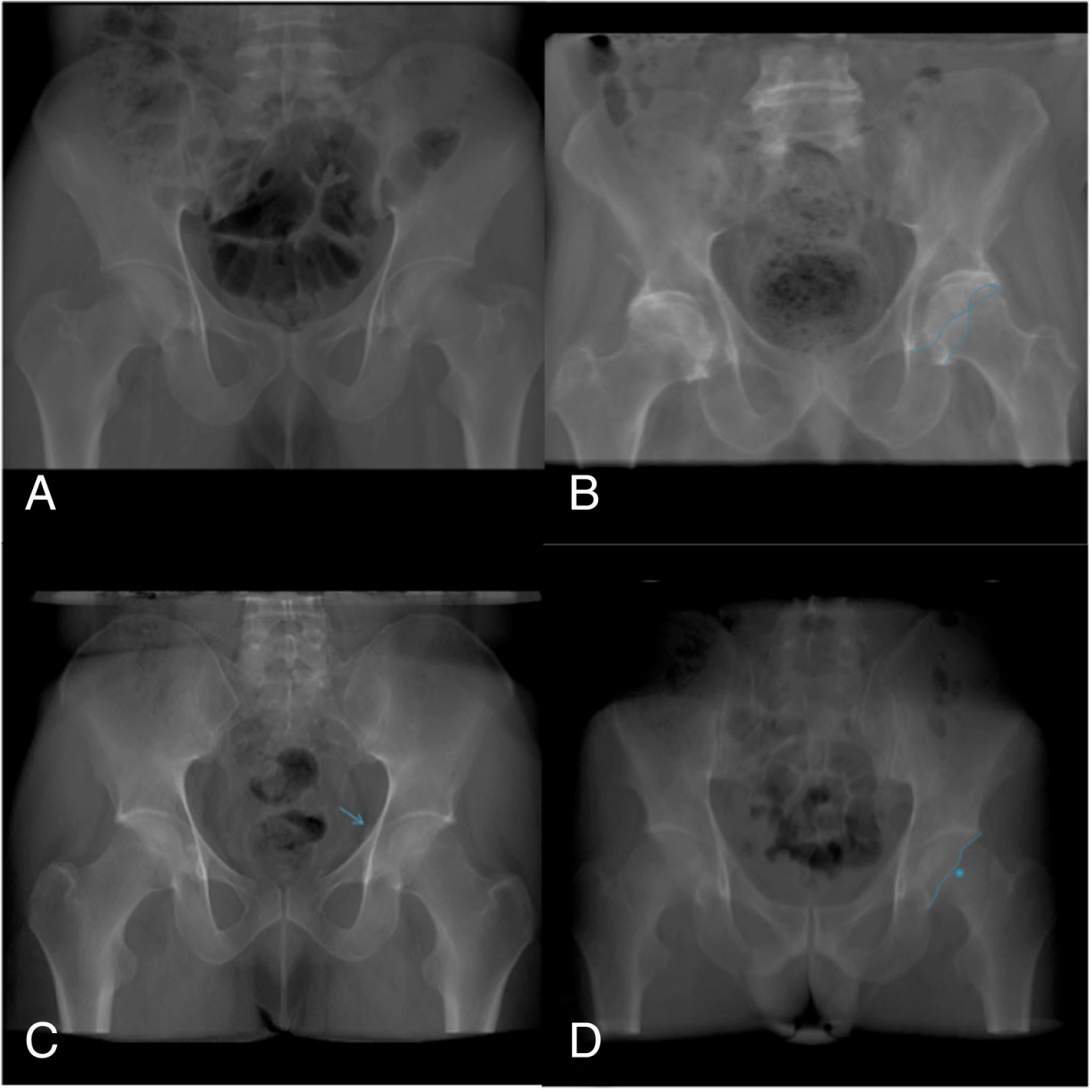
**a** Pelvis with no radiographical signs of acetabular retroversion. **b** Antero-posterior view, showing the cross-over sign on the left side. **c** Pelvis with the prominence of the ischial spine sign. The arrow indicates the ischial spine projecting into the lower pelvis on the left side. **d** Showing the posterior wall sign in the left hip. The posterior wall projects more medially then the center of the femoral head

The cross-over sign is considered positive when the anterior edge of the acetabulum is no longer medially to the posterior edge. Acetabular retroversion typically starts by being accentuated at the cranial part of the anterior edge. On antero-posterior radiographs this is seen as an intersection of the two edges [10].

### Prominence of the ischial spine sign (Fig. 2c)

The prominence of the ischial spine sign is positive when the ischial spine is situated medially to the pelvic ring, projecting into the small pelvis.

### Posterior wall sign (Fig. 2d)

The posterior wall sign is described as the phenomenon when the posterior acetabular edge no longer lies laterally in respect to the center of the femoral head on a.p. radiographs.

### Statistical analysis

All data was recorded in an Excel database (Microsoft Corp., Washington, DC, USA) and exported to SPSS 22.0 (SPSS Inc., Chicago, IL, USA) for statistical analysis. Differences in means were assessed using a non-parametric Mann–Whitney-Test.

### Ethics

For radiological measurements of the pelvis and the spine on humans, an application for ethical approval was summited to the regional ethics commission (Kantonale Ethikkommission Zürich). This application was approved (KEK-ZH-Nr.2011-0507).

Because of the nature of the study, which included solely clinical data collection, the need to obtain informed written consent was waived.

## Results

PI was assessed in 589 individuals (398 males and 191 females). Mean PI was 50.9° with a standard deviation of 11.0°. There was no significant difference between male and female patients. After rendering a 2D a.p. view from CT data sets and adding the few conventional radiographs 477 hips could be evaluated for COS and 478 hips for PWS and PRISS. Of the hips evaluated for the COS, 18 % were found to have the sign present, with the PWS 4 % and with the PRISS 3 % respectively. When evaluated for differences in mean pelvic incidence in regard to the presence or absence of the cross-over sign, there was no significant correlation between PI and the appearance of a cross-over sign. For PWS and PRISS, there was a significant difference in mean PI between parameter positive and parameter negative hips (PWS *p* = .021; PRISS *p* = .002). Mean PI was 45.2° when the posterior wall sign was present, in the presence of the prominence of the ischial spine sign it was 40.7° (Table 1).

**Table 1:** Shows the different mean PI for hips with positive and negative parameters for acetabular retroversion. n (+/−), number of hips with positive or negative parameter; PI (+/−): respectively PI of positive and negative hips. STD is the standard deviation. Sig. is the *p*-value indicating whether the difference in mean PI was significant or not

	n	n(+)	PI/STD(+)	n(−)	PI/STD(−)	sig.
COS	477	86	48.9/10.5	391	50.5/10.6	.170
PWS	478	18	45.2/12.2	452	50.5/10.5	.021
PRISS	478	12	40.7/8.1	459	50.6/10.5	.002

## Discussion

A previous study showed that pelvic tilt positively correlated with increased acetabular coverage [23]. Pelvic tilt, however, is a positionally variable parameter. To our knowledge, no work has been done on the relationship of pelvic incidence as a positionally non-altering parameter and acetabular coverage. A high PI leads to a higher lumbar lordosis in most cases, and thus, to a higher maneuverability around the bicoxofemoral axis [21]. We postulated that a lower adaptability within the spinopelvic system and a decreased potential to retrovertetly rotate the pelvis around the bicoxofemoral axis in particular, would lead to degenerative changes within the hip as well as to acetabular retroversion. This would indicate an inverse correlation between PI and the occurrence of parameters for acetabular retroversion; in other words, lower PI values of parameter positive hips. Prevalence of COS positive hips was 18 %. It is hard to compare this to other studies as COS tends to be interpreted quite variably. In respect to PRISS and PWS the prevalence was lower than previously reported [12]. This might be due to the manner in which we assessed hips for COS, which is elaborated further down.

Indeed we could observe that where PRISS and PWS were present the PI values were significantly lower than hips where those signs were absent. This suggests that a decrease in PI and acetabular retroversion are linked. However, we would refrain from postulating a direct causal dependency, as correlations are not easily interpreted within the complex spinopelvic system. The failure to produce any significant results with the COS parameter has to be put in perspective, as COS is the parameter most vulnerable to decrease in resolution in evaluated radiographs. This was the case when rendering 2D a.p. view of the 3D CT data set. Another fact that could be the reason for not attaining a significant result with the cross-over sign in this study, was that all signs of a cross-over, even if they was very cranially were counted as hips positive for COS. The problem is that there is to date no accepted cut off in terms of cross-over ratio that differentiates between a clinically significant cross-over and a cross-over that might be visible at the very top of the acetabular dome but has no clinical impact. This leads us to the limitations of this study. First, although the a.p. reconstruction from CT data sets is an elegant way to look at conventional parameters for acetabular retroversion if no conventional imaging is available, images gained from such renderings are inferior in resolution to conventional anterio-posterior radiographs. As mentioned above, this would have the greatest impact when assessing for cross-over sign positivity, as intersections that lie very cranially could be missed. In 4 % of the hips assessed for COS it was not possible to determine whether the radiological sign was present or not. Secondly, there was no interobserver control, meaning that although different parameters were measured by different people, the same parameter was only measured by one person. Any further studies should look at the relationship between COS and PI. It could very well be that if a cut off for the cross-over ratio was chosen that would select hips with a significant degree of retroversion, a significant difference in pelvic incidence would be found. It would also be interesting to look at the correlation between the degree in retroversion measured directly from CT scans as a continuous value and the pelvic incidence.

## Conclusion

Hips where PRISS or PWS were present showed significantly lower PI values than hips where those radiological sign were absent. This suggests that lower PI values correlate with retroversion of the acetabular dome. Further research should focus on the correlation between the degree of retroversion and the PI values.

## Abbreviations

PI: pelvic incidence
COS: cross-over sign
PWS: posterior-wall sign
PRISS: prominence of the ischial spine sign
a.p: anterio-posterior
